# Analysis of Phenolic Acids and Flavonoids in Rabbiteye Blueberry Leaves by UPLC-MS/MS and Preparation of Nanoemulsions and Extracts for Improving Antiaging Effects in Mice

**DOI:** 10.3390/foods12101942

**Published:** 2023-05-10

**Authors:** Hsin-Rong Yu, Bing-Huei Chen

**Affiliations:** 1 Department of Food Science, Fu Jen Catholic University, New Taipei City 242062, Taiwan; mollysjyu@gmail.com; 2 Department of Nutrition, China Medical University, Taichung 40402, Taiwan

**Keywords:** rabbiteye blueberry leaves, phenolic acid, flavonoid, nanoemulsion, mice aging

## Abstract

Rabbiteye blueberry leaves, a waste produced after harvest of blueberry, are rich in polyphenols. This study aims to analyze phenolic acids and flavonoids in blueberry leaves by UPLC-MS/MS and prepare nanoemulsions for determining anti-aging activity in mice. Overall, 30% ethanol was the most suitable extraction solvent for total phenolic acids and total flavonoids. A total of four phenolic acids and four flavonoids were separated within seven minutes for further identification and quantitation by UPLC-MS/MS in selective reaction monitoring (SRM) mode, with 3-*O*-caffeoylquinic acid being present in the highest amount (6474.2 μg/g), followed by quercetin-3-*O*-galactoside (1943.9 μg/g), quercetin-3-*O*-rutinoside (1036.6 μg/g), quercetin-3-*O*-glucoside (867.2 μg/g), 5-*O*-caffeoylquinic acid (815.8 μg/g), kaempferol-3-*O*-glucoside (309.7 μg/g), 3,5-dicaffeoylquinic acid (195.3 μg/g), and 4,5-dicaffeoylquinic acid (60.8 μg/g). The blueberry nanoemulsion was prepared by using an appropriate ratio of soybean oil, Tween 80, glycerol, ethanol, and water at 1.2%, 8%, 2%, 2%, and 86.8%, respectively, and mixing with dried blueberry extract, with the mean particle size and zeta potential being 16 nm and −54 mV, respectively. A high stability was observed during storage of nanoemulsion for 90 days at 4 °C and heated at 100 °C for 2 h. An animal study revealed that this nanoemulsion could elevate dopamine content in mice brain as well as superoxide dismutase, glutathione peroxidase, and catalase activities in mice liver while reducing the contents of malondialdehyde and protein carbonyl in mice brains. Collectively, the high-dose nanoemulsion possessed the highest efficiency in improving mice aging with a promising potential for development into a health food.

## 1. Introduction

Rabbiteye blueberry (*Vaccinium virgatum*), an evergreen shrub of the Ericaceae plant group rich in phenolic compounds, is known to possess a delicate flavor and high nutritive value. In addition, many reports have shown that blueberry has several important biological functions such as anti-inflammation [1], anti-obesity [2], anti-diabetes [3], anti-tumor [4], and protection of cardiovascular disease [5], all of which can be due to the presence of bioactive compounds, including anthocyanins, flavonoids, and phenolic acids. Compared to blueberry fruits, blueberry leaves were reported to contain higher amounts of phenolic compounds. For instance, Li et al. [6] showed that the total phenolic content (TPC) and flavonoid content (TFC) were higher in the ethanolic extracts of blueberry leaves (339.10 mg gallic acid equivalent (GAE)/g and 198.10 mg quercetin equivalent (QE)/g dry weight) than in blueberry fruits (216.43 mg GAE/g and 115.55 mg QE/g dry weight). Likewise, more recently, Debnath-Canning et al. [7] reported that phenolic compounds were present in higher amounts in 4 different cultivar varieties of blueberry leaves than in fruits with the TPC and TFC respectively ranging from 111.71–184.99 mg GAE/g and 3.90–8.19 μmol catechin equivalent (CE)/g fresh weight in leaves as well as 4.72–6.60 mg GAE/g and 0.09–0.17 μmol CE/g fresh weight in fruits. It was also reported that the polyphenol content in blueberry leaves was 3-fold higher than that in blueberry fruit [8]. Specifically, Rabbiteye blueberry leaves were shown to be rich in chlorogenic acid, quercetin, anthocyanin, and proanthocyanin [9]. Furthermore, many previous studies have reported that the intake of blueberry leaves can be protective against chronic diseases such as cataracts [10], diabetes [11], and hypertension [12]. However, blueberry leaves are often discarded during the harvesting and processing of blueberry products. Thus, the use of blueberry leaves as a raw material can be advantageous for the production of health foods.

However, many bioactive compounds such as polyphenols suffer the major drawback of low stability and bioavailability in vivo. To overcome this issue, many encapsulation techniques such as nanoemulsion have been developed to enhance stability and bioavailability of bioactive compounds in vivo [13]. For instance, Tran et al. [14] prepared a quercetin nanoemulsion system composed of castor oil, Tween 80, cremophor RH 40, and PEG 400 for elevation of solubility and bioavailability in vivo. In another study, to increase the stability, aqueous solubility, and skin permeation of astaxanthin, Hong et al. [15] developed a carboxymethyl chitosan functionalized nanoemulsion composed of Cremophor EL and Labrafil M 1944 CS.

Accordingly, the aging theory can be classified into genetic preset and stochastic destruction, with the former showing alteration of preset genetic structure or genetic expression, leading to aging, and the latter showing stochastic destruction of large molecules such as nucleic acid and protein in vivo caused by free radical formation, oxidation or glycation [16]. Specifically, free radicals can be formed through normal metabolism or defense mechanism in vivo when invaded by microorganisms or virus. Additionally, free radicals can be produced following UV radiation, smoking, air pollution or oxidation of polyunsaturated fatty acid [17]. Thus, oxidative stress can occur when the number of free radicals produced in vivo exceeds that which can be eliminated through the defense mechanism of humans, leading to the occurrence of chronic disease and aging [18]. In addition, the genetic factor and damage to mitochondrial DNA caused by reactive oxygen species (ROS) can also lead to aging [19]. Therefore, in fairness, aging is a participation process of multiple factors, with heredity and various environmental factors playing a vital role. In addition to regulation of genetic factors, external factors such as environment and dietary habits can also affect the aging rate. It has been well-established that the ROS accumulation can destruct biological molecules such as DNA, protein, and fat, resulting in cell apoptosis [20]. Moreover, ROS is mainly produced in the complex III of mitochondria within cells and complex I in brain and heart organs, both of which can lead to severe damage of mitochondrial DNA [20]. Thus, the measurement of mtDNA oxidative damage has become an important index of aging. Furthermore, of the various body organs, the brain is the most vulnerable to aging-related oxidative stress for the following reasons: (1) the brain consumes about 20% of the total oxygen, (2) the brain contains high amounts of polyunsaturated fatty acids which can be prone to oxidation, (3) the accumulation of irons in brain can facilitate free radical production, and (4) the shortage of iron-binding proteins such as ferritin [21,22]. Therefore, the neurodegenerative diseases caused by oxidative damage such as Alzheimer’s disease and Parkinson’s disease can be directly associated with the production of ROS and free radicals [23]. In addition to the brain, some other organs such as heart, liver, kidney, and bone can also be affected by aging due to oxidative stress and damage [24].

This study aimed to extract phenolic acids and flavonoids from rabbiteye blueberry leaves for the preparation of extracts and nanoemulsions and for the comparison of their anti-aging effects in mice. Based on the accession number 251464, the Rabbiteye blueberry *leaf (Vaccinium virgatum)* was identified by being deposited in the herbarium of Taiwan Forestry Research Institute (TAIF, Taipei, Taiwan).

## 2. Materials and Methods

### 2.1. Materials

Rabbiteye blueberry leaves from twenty different cultivars, including Gulf coast1, Sharp blue, Climax, Misty, O’Neal, Gloria, Fuku, Powderblue, Gwl coast, Alice, GGM, Tifblue, Sunshine blue, NTU 042, NTU 03, Gulf coast2, Onel, Sunshine, NTU 104 (fresh leaves), and NTU 104 (withered leaves), were provided by Taiwan Flower Biotechnology Co. (Taipei, Taiwan). Following washing and freeze-drying (−45 °C, 125 mT) for four days, approximately 545 g of blueberry leaves was collected and ground into a powder for vacuum sealing and was stored at −20 °C for subsequent experiments.

Phenolic acid standards, including 5-*O*-caffeoylquinic acid (neochlorogenic acid), 3,5-dicaffeoylquinic acid (isochlorogenic acid A) and 4,5-dicaffeoylquinic acid (isochlorogenic acid C), were procured from Biopurity Phytochemicals Ltd. (Chendu, China), while 3-*O*-caffeoylquinic acid (chlorogenic acid) was from Tauto Biotech Co. (Shanghai, China), with the purity of all the standards being higher than 98%. Flavonoid standards, including quercetin-3-*O*-glucoside (isoquercetin), quercetin-3-*O*-galactoside (hyperoside), and kaempferol-3-*O*-glucose (astragalin), were purchased from Biopurity Phytochemicals Ltd. (Chendu, China), with the purity being all higher than 98%, while quercetin-3-*O*-rutinoside (rutin) and (-)-epigallocatechin gallate (EGCG) were from Sigma-Aldrich Co. (St. Louis, MO, USA) with the purity being higher than 94%.

Solvents such as acetonitrile and ethanol were from Merck Co. (Darmstadt, Germany) and Sigma-Aldrich Co., respectively. Yu Pa Co. (Taipei, Taiwan) provided both Tween 80 and glycerol used for the nanoemulsion preparation, while Sigma-Aldrich supplied both D-galactose and potassium dihydrogen phosphate. Milli-Q water purification system supplied the deionized water for all dilution purposes (Millipore Co., Bedford, MA, USA).

The assay kits, including superoxide dismutase, TBARs, glutathione peroxidase, protein carbonyl, and catalase, were from Cayman Chemicals Co. (Ann Arbor, MI, USA). The ELISA kit for determining mouse dopamine was from CUSABIO Co. (Houston, TX, USA). A total of 36 6-week-old C57BL/6 male mice was obtained from National Experimental Animal Center (Taipei, Taiwan). After pre-feeding for two weeks, a 12-week animal experiment was conducted.

### 2.2. Determination of Total Phenolic Content (TPC) and Total Flavonoid Content (TFC)

Initially, for comparison of extraction efficiency, 0.5 g of rabbiteye blueberry leaf powder was mixed with 30 mL of 30%, 50%, or 70% ethanol (*n* = 3), followed by shaking each mixture in a 60 °C water bath for 3 h, centrifuging at 1789× *g* for 30 min (25 °C), collecting the supernatant, filtering with a 0.6-μm filter paper, evaporating to dryness in a N-1200A model Eyela rotary evaporator (Sunway Scientific Corporation, Taipei, Taiwan) at 40 °C, dissolving in 10 mL of deionized water, and filtering with a 0.22-μm polyvinylidene fluoride (PVDF) membrane filter for the analysis of TPC and TFC.

A method based on Kao et al. [25] was used to determine TPC in rabbiteye blueberry leaves. In brief, 200 μL of Folin-Ciocalteu reagent was added separately to five different concentrations of gallic acid standard (50–440 μg/mL). After mixing for homogeneity, the mixture was left in dark for 5 min, and 1 mL of sodium carbonate (15%) was added and allowed to react at 25 °C for 1 h for measuring the absorbance at 750 nm using a VersaMax ELISA microplate reader (Molecular Devices, Sunnyvale, CA, USA). Next, a 50 mL of sample extract was collected, and the absorbance was measured at 750 nm for determination of TPC based on the gallic acid standard curve.

Similarly, a method based on Kao et al. [25] was used to determine TFC in rabbiteye blueberry leaves. Initially, 30 μL of 5% sodium nitrite solution was mixed separately with 200 μL of 6 concentrations of quercetin standard (25–400 μg/mL) and was allowed to stand for 5 min. Following that, 60 μL of aluminum chloride (10%) was added and, after 5 min, 300 μL of sodium hydroxide (1 M) and 200 μL of chloroform were added, followed by centrifuging and collecting the supernatant for measuring absorbance at 510 nm in a VersaMax ELISA microplate reader. By using the linear regression equation of quercetin standard curve, the TFC in rabbiteye blueberry leaves was determined by measuring the absorbance of sample extract (200 μL) at 510 nm.

### 2.3. Analysis of Individual Phenolic Acid and Flavonoid by UPLC-MS/MS

Following the comparison of extraction efficiency of various ethanol proportions, 30% ethanol was chosen for extraction and for subsequent UPLC-MS/MS analysis. In brief, 0.5 g sample was mixed with 30 mL of 30% ethanol and shaken in a 60 °C water bath for 3 h, after which the mixture was centrifuged at 1789× *g* (25 °C) for 30 min and the supernatant was collected for filtering through a filter paper, followed by evaporating to dryness in an Eyela rotary evaporator at 40 °C and dissolving in 10 mL deionized water. One mL was collected, evaporated to dryness, dissolved in 1 mL of 30% ethanol, and the internal standard EGCG was added (10 μg/mL). After filtration through a 0.22-μm PVDF membrane filter, a 5 μL sample was injected for UPLC-MS/MS analysis.

A method based on Chen et al. [26] was modified and used for the determination of individual phenolic acid and flavonoid compounds in rabbiteye blueberry leaf powder. By using a Waters Acquity UPLC BEH C18 column (100 × 2.1 mm ID, particle size 1.7 μm) coupled with a triple quadrupole tandem mass spectrometer (QqQ) (Dionex Ultimate 3000 Open Sampler XRS System (Thermo Fisher Scientific Co., San Jose, CA, USA) in electrospray ionization mode, a total of four phenolic acids and four flavonoids were separated within 7 min with flow rate at 0.3 mL/min, column temperature at 30 °C, and the following gradient mobile phase: 0.1% formic acid solution (A) and 100% acetonitrile (B) with 100% A and 0% B in the beginning, changed to 20% B at 4 min, 30% B at 9 min, and 100% B at 14 min.

For identification by UPLC-MS/MS, the selected reaction monitoring (SRM) with negative ion mode was used with ion transfer tube temperature, vaporizer temperature, sweep gas flow rate, sheath gas flow rate, auxiliary gas flow rate, and spray voltage at 329 °C, 279 °C, 0 arbitrary units (a.u.), 38 a.u., 12 a.u., and 3000 V, respectively. Following that, the various phenolic acids and flavonoids in rabbiteye blueberry leaves were identified by comparison of retention time, precursor ion, and product ion of unknown peaks with that of reference standards.

For the determination of limit of detection (LOD) and limit of quantitation (LOQ), a total of 13 concentrations of individual phenolic acid and flavonoid standards (0.0001–0.1 μg/mL) were prepared separately. After the injection of each concentration into UPLC-MS/MS three times, the standard curve was obtained by plotting concentration against peak area. Both slope (S) and the standard deviation (δ) were obtained to calculate LOD and LOQ by using the following formula:

$$\mathrm{LOD}=\frac{3.3\times \;\delta }{S}$$

LOQ = 3 × LOD

The intra-day variability (repeatability) was obtained by analyzing individual phenolic acid and flavonoid in rabbiteye blueberry leaves with the internal standard EGCG (10 μg/mL) three times a day with each time analyzed in triplicate for a total of nine analyses. Likewise, the inter-day variability (intermediate precision) was determined using the same approach as the intra-day variability, with the exception that blueberry leaves were analyzed on three consecutive days with triplicate analyses each day for a total of nine analyses.

For recovery determination, two concentrations of various phenolic acid and flavonoid standards, including 3-*O*-caffeoylquinic acid (350 and 3500 μg), 5-*O*-caffeoylquinic acid (45 and 450 μg), 3,5-dicaffeoylquinic acid (10.5 and 105 μg), 4,5-dicaffeoylquinic acid (3.5 and 35 μg), quercetin-3-*O*-galactoside (110 and 1100 μg), quercetin-3-*O*-glucoside (42.5 and 425 μg), quercetin-3-*O*-rutinoside (55 and 550 μg), and kaempferol-3-*O*-glucoside (17.5 and 175 μg), were added to blueberry leaves separately for extraction and quantitation by UPLC-MS/MS. The recovery was calculated by using the following formula:

$$\mathrm{Recovery}\;(\%)=\frac{\mathrm{amount}\;\mathrm{found}\;\mathrm{after}\;\mathrm{UPLC}\;-\;\mathrm{original}\;\mathrm{amount}\;\mathrm{before}\;\mathrm{UPLC}}{\mathrm{amount}\;\mathrm{spiked}\;\mathrm{before}\;\mathrm{UPLC}}\;\times \;100$$

### 2.4. Quantitation of Individual Phenolic Acid and Flavonoid

A method based on Huang and Chen [27] was modified and used for the quantitation of individual phenolic acid and flavonoid compounds in rabbiteye blueberry leaf powder. A total of six concentrations (0.01, 0.1, 0.5, 1, 5, and 10 μg/mL) were each prepared for each phenolic acid and flavonoid standard and were then mixed with EGCG at 10 μg/mL. After injection into UPLC-MS/MS, the standard curve of individual phenolic acid and flavonoid was prepared by plotting concentration ratio (standard versus internal standard) against area ratio (standard versus internal standard) to obtain both linear regression equations and R^2^. The individual phenolic acid and flavonoid content in rabbiteye blueberry leaves was determined using the formula shown below:

$$\mathrm{Phenolic}\;\mathrm{acid}/\mathrm{Flavonoid}\;(\mu g/g)=\;\left(\frac{A_{s}}{A_{i}}-b\right)\times \frac{1}{a}\times C_{i}\times V\times \mathrm{DF}\times \frac{1}{\mathrm{recovery}}\times W_{s}$$

where A_s_: peak area of phenolic acid or flavonoid; A_i_: peak area of internal standard (EGCG); a: slope of the standard curve; b: intercept of the standard curve; C_i_: concentration of internal standard (μg/mL); V: final volume of sample extract (mL); DF: dilution factor; W_s_: weight of the sample (g).

### 2.5. Preparation of Rabbiteye Blueberry Leaf Nanoemulsion

Initially, 0.5 g of rabbiteye blueberry leaf powder from NTU 104 (fresh leaves) with the highest phenolic acid and flavonoid contents was extracted with 30% ethanol by adopting the same procedure described under Section 2.3. A method based on Chen et al. [26] was used for the preparation of nanoemulsion with slight modification. 10 mL of rabbiteye blueberry leaf sample extract containing four phenolic acids at 5 mg/mL was prepared and evaporated to dryness under nitrogen, followed by adding 0.12 g (1.2%) of soybean oil, 0.2 g (2%) of 30% ethanol, 0.8 g (8%) of Tween 80, and 0.2 g (2%) of glycerol with intermittent stirring after each ingredient addition, and finally 8.68 g (86.8%) of deionized water was added, stirred thoroughly, and was sonicated for 30 min for successful preparation of a transparent nanoemulsion.

### 2.6. Determination of Nanoemulsion Characteristics

#### 2.6.1. Particle Size and Polydispersity Index

A 100 μL sample of nanoemulsion was diluted 50-fold with potassium dihydrogen phosphate (pH 5.3–5.5), followed by placing in a styrene tube for measurement of mean particle size and polydispersity index (PDI) by a dynamic light scattering instrument (90 plus particle size analyzer) from Brookhaven Instruments (Holtsville, NY, USA) [28].

#### 2.6.2. Zeta Potential

The zeta potential was determined by 200-fold dilution of 100-μL nanoemulsion sample with deionized water and measurement of zeta potential by a SZ-100 Horiba analyzer (Kyoto, Japan) [27].

#### 2.6.3. Transmission Electron Microscopy

After diluting 100 μL of nanoemulsion sample with deionized water, 20 μL was placed on a copper grid, allowed to stand for 90 s, and the surplus sample was removed by a filter paper. For negative staining, uranyl acetate was added, allowed to stand for 2 min, and the surplus uranyl acetae was removed using a filter paper and was then dried in a desiccator overnight for capturing images under a JEM-1400 JEOL TEM instrument (Tokyo, Japan) by magnifying up to 2.5 × 10^5^ times [28]. 

#### 2.6.4. Encapsulation Efficiency

For determination of encapsulation efficiency, 100 μL of nanoemulsion sample was diluted 10 times with deionized water. The sample was then poured into a centrifuge tube equipped with a 3 kDa dialysis membrane, followed by centrifuging at 13,800× *g* (25 °C) for 20 min, collecting the bottom layer for determination of four phenolic acids by UPLC-MS/MS. The encapsulation efficiency of four phenolic acids was calculated using the following formula [26]:

$$\mathrm{Encapsulation}\;\mathrm{efficiency}=\frac{\mathrm{total}\;\mathrm{amount}\;\mathrm{of}\;4\;\mathrm{phenolic}\;\mathrm{acids}\;-\;\mathrm{amount}\;\mathrm{of}\;4\;\mathrm{free}\;\mathrm{phenolic}\;\mathrm{acids}}{\mathrm{total}\;\mathrm{amount}\;\mathrm{of}\;4\;\mathrm{phenolic}\;\mathrm{acids}}\;\times \;100$$

### 2.7. Stability of Nanoemulsion

The stability of the nanoemulsion was determined by storing 10 mL of sample at 4 °C for 90 days and a portion was collected at regular intervals for determination of mean particle size, PDI and zeta potential. For heating stability, 200 µL of nanoemulsion sample was placed in a water bath with temperature controlled at 40, 60, 80, or 100 °C, as well as heating time length at 0.5, 1.0, 1.5, or 2 h. At the end of each time interval, the sample was collected for the determination of mean particle size, PDI, and zeta potential [27].

### 2.8. Animal Experiment

A total of 36 6-week-old C57BL/6 male mice were raised in Fu Jen University Experimental Animal Center with temperature at 21 ± 2 °C, relative humidity at 55 ± 10%, and light circle for 12 h. Both animal handling and experimental procedures were based on the guidelines set by Fu Jen Catholic University Experimental Animal Care and Use Committee (approval number A 10901). 

Both diet and water were provided ad libitum. After a two-week adaptation period, all the 8-week-old mice with a body weight of about 27 g each were subjected to a 12-week animal experiment by dividing into six groups with six mice each:

1. Control group (C): mice were provided with sterilized water by subcutaneous injection once a day for the first 6 weeks and then tube feeding with sterilized water, starting at the 7th week once a day for another 6 weeks.
2. Induction group (D): mice were provided with D-galactose at a dose of 300 mg/kg bw by subcutaneous injection once a day for the first 6 weeks and then tube feeding with sterilized water starting at the 7th week once a day for another 6 weeks.
3. Low-dose extract (LE): mice were provided with D-galactose at a dose of 300 mg/kg bw by subcutaneous injection once a day for the first 6 weeks and then tube feeding with blueberry extract (dissolved in 0.2 mL of deionized water) at a dose of 8 mg/kg bw containing 0.2 mg of four phenolic acids starting at the 7th week once a day for another 6 weeks.
4. High-dose extract (HE): mice were provided with D-galactose at a dose of 300 mg/kg bw by subcutaneous injection once a day for the first 6 weeks and then tube feeding with blueberry extract (dissolved in 0.2 mL of deionized water) at a dose of 40 mg/kg bw containing 1 mg of four phenolic acids starting at the 7th week once a day for another 6 weeks.
5. Low-dose nanoemulsion (LN): mice were provided with D-galactose at a dose of 300 mg/kg bw by subcutaneous injection once a day for the first 6 weeks and then tube feeding with 0.2 mL of nanoemulsion at a dose of 8 mg/kg bw containing 0.2 mg of four phenolic acids starting at the 7th week once a day for another 6 weeks.
6. High-dose nanoemulsion (HN): mice were provided with D-galactose at a dose of 300 mg/kg bw by subcutaneous injection once a day for the first 6 weeks and then tube feeding with 0.2 mL of nanoemulsion at a dose of 40 mg/kg bw containing 1 mg of four phenolic acids starting at the 7th week once a day for another 6 weeks.

After feeding for 12 weeks, all the mice were euthanized with carbon dioxide, and both brain and liver were collected for weight measurement and then stored at −80 °C for subsequent analyses.

### 2.9. Determination of Activities of Superoxide Dismutase (SOD), Glutathione Peroxidase (GSH-Px), and Catalase (CAT) in Mice Liver

By adopting the instructions provided by the assay kits from Cayman Chemical Co. (Ann Arbor, MI, USA), the activities of SOD, GSH-Px, and CAT antioxidant enzymes were determined in mice liver through measuring absorbance using VersaMax ELISA microplate reader.

In brief, a total of six concentrations, including 0.005, 0.010, 0.020, 0.030, 0.040, and 0.050 U/mL, were prepared for the bovine erythrocyte SOD (Cu/Zn) standard, after which a 10 μL standard from each concentration and sample was collected separately and added to a 96-well plate, followed by adding 200 μL of radical detector and mixing homogeneously. Following that, 20 μL of xanthine oxidase was added to initiate the reaction, and the absorbance at 450 nm was measured following shaking at room temperature for 30 min, while the SOD activity of each sample was calculated based on the standard curve [26].

For GSH-Px activity, a 20 μL of GSH-Px standard and sample were added to a 96-well plate separately and then 50 μL of assay buffer, 50 μL of co-substrate mixture, and 50 μL of NADPH were added and mixed homogeneously, followed by adding 20 μL of cumene hydroperoxide to initiate the reaction. Following shaking for a few seconds at room temperature, the absorbance was measured at 340 nm once every minute for five times in which the values at two time points were selected to calculate the GSH-Px activity of each sample [26].

For CAT activity, a total of six concentrations, including 5, 15, 30, 45, 60, and 75 μM, were prepared for the formaldehyde standard, after which a 20 μL standard from each concentration and sample were added to a 96-well plate separately and then 100 μL of the assay buffer and 30 μL of methanol were added. After mixing homogeneously, 20 μL of hydrogen peroxide was added to initiate the reaction, followed by shaking for 20 min at room temperature, adding 30 μL of potassium hydroxide to terminate the reaction, adding 30 μL of catalase purpald, shaking at room temperature for 10 min, and then adding 10 μL of potassium periodate. Following standing for 5 min, the absorbance was measured at 540 nm for calculation of the CAT activity of each sample based on the standard curve [26].

### 2.10. Determination of Dopamine, Malondialdehyde (MDA), and Protein Carbonyl (PC) Contents in Mice Brain

The contents of dopamine, MDA, and PC in mice brains were determined by following the instructions in the assay kits obtained from Cayman Chemical Co (Ann Arbor, MI, USA), while that of dopamine from CUSABIO Co. (Houston, TX, USA) measured the absorbance in a VersaMax ELISA microplate reader.

Briefly, a total of seven concentrations, including 1.562, 3.125, 6.25, 12.5, 25, 50, and 100 ng/mL, were prepared for the dopamine standard, after which a 50 μL standard from each concentration and sample was collected separately, followed by adding 50 μL of biotin-labeled antibody and 50 μL of horseradish peroxidase conjugate. After mixing homogeneously at 37 °C for 1 h, this mixture was washed with wash buffer three times, followed by adding 50 μL of substrate A and 50 μL of substrate B, mixing thoroughly, reacting at 37 °C for 15 min, and adding 50 μL of stop solution to terminate the reaction. The absorbance at 450 nm was measured for calculation of the dopamine content in each sample based on the standard curve [29].

For MDA content, a total of seven concentrations, including 0.625, 1.25, 2.5, 5, 10, 25, and 50 μM, were prepared for the MDA standard, after which a 100 μL standard from each concentration and sample was collected separately, followed by adding 100 μL of sodium dodecyl sulfate (SDS) and 4 mL of coloring agent, placing in a water bath (100 °C) for reacting for 1 h, cooling on ice for 10 min, centrifuging at 1600× *g* for 10 min (4 °C), collecting the supernatant, and adding to a 96-well plate. The absorbance was measured at 535 nm for the calculation of the MDA content in samples based on the standard curve [26].

For PC content, a 200 μL of sample was poured into a sample centrifuge tube and control centrifuge tube separately, followed by adding 800 μL of DNPH to the former and 800 μL of hydrochloric acid (2.5 M) to the latter. Both tubes were stood in the dark for 1 h and then 1 mL of 20% trichloroacetic acid (TCA) was added. After mixing homogeneously, this mixture was placed on ice for 5 min and then centrifuged at 10,000× *g* for 10 min (4 °C). The supernatant was removed and 1 mL of 10% TCA was added to the protein pellet, homogenized again, placed on ice for 5 min, and centrifuged at 10,000× *g* for 10 min (4 °C). The supernatant was removed again, and 1 mL of ethanol/ethyl acetate was added to the protein pellet, homogenized, centrifuged at 10,000× *g* for 10 min (4 °C), and the supernatant removed. One mL of ethanol/ethyl acetate was added twice, followed by adding 500 μL of guanidine hydrochloride to the protein pellet, centrifuging at 10,000× *g* for 10 min (4 °C), collecting the supernatant, and measuring absorbance at 370 nm in a 96-well plate for calculation of PC contents in samples [30].

### 2.11. Statistical Analysis

Statistical analysis of all the experimental data was done by using statistical analysis system (SAS) software [31] for analysis of variance by ANOVA and Duncan’s multiple range test for significant difference of mean values (*p* < 0.05).

## 3. Results and Discussion

### 3.1. Effect of Solvent Variety on Total Phenolic Content (TPC) and Total Flavonoid Content (TFC) in Rabbiteye Blueberry Leaves

Owing to the more hydrophilic nature of polyphenols, they are commonly extracted from plant products by solvents such as methanol, ethanol, acetonitrile, and acetone alone or in combination with water [27,28]. However, in this study, an aqueous solution of ethanol was used as the extraction solvent, as it is relatively safer (less toxic) and used as a universal solvent with a polarity index of 5.2 and dielectric constant of 24.55 capable of solubilizing both polar and less polar polyphenols [32]. Table 1 shows the effect of different ethanol proportions on TPC and TFC in rabbiteye blueberry leaves. The highest TPC, expressed as gallic acid equivalent (mg/g), was shown for 50% EtOH, followed by 30% EtOH and 70% EtOH. However, the difference in TPC between 30% EtOH and 50% EtOH was not significant (*p* > 0.05). For the TFC expressed as quercetin equivalent (mg/g), 30% EtOH produced the highest yield, followed by 50% EtOH and 70% EtOH. Furthermore, a lesser volume of ethanol used in the preparation of blueberry leaf extract when compared to 50% ethanol favors the green sustainable strategy of reducing solvent usage. Thus, 30% EtOH was selected as the extraction solvent for subsequent experiments.

In several previous studies, Routray and Orsat [33] reported that a higher TPC from Bluetta blueberry leaves was obtained by 30% EtOH than that by 15% EtOH. Likewise, compared to 95% EtOH, 50% EtOH was shown to result in a higher yield of TPC in blueberry leaves [34]. Additionally, an average of TPC (137.61 mg/g) and TFC (249.88 mg/g) from 73 different cultivars of rabbiteye blueberry leaves in southeastern China was reported by Wu et al. [35]. Apparently, the difference in TPC and TFC in blueberry leaves can be affected by species, harvest time, and the extraction method.

Table 2 shows TPC and TFC in different cultivars of rabbiteye blueberry leaves used in this study. A total of 20 cultivars were collected in this study, with the cultivar NTU 104 showing the highest TPC (136.09 mg/g) and a medium TFC (36.25 mg/g). Therefore, the cultivar NTU 104 (fresh leaves) was selected for subsequent experiments.

### 3.2. Analysis of Phenolic Acids and Flavonoids in Rabbiteye Blueberry Leaves by UPLC-MS/MS

**Figure 1:**
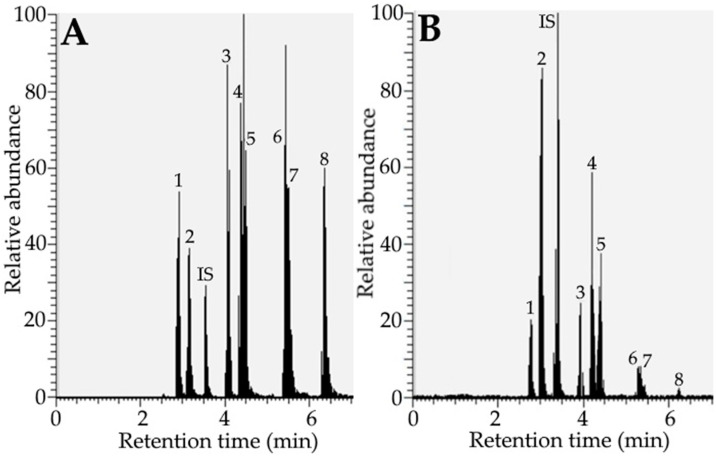
UPLC-MS/MS-TIC chromatogram of phenolic acids and flavonoids from standards mixture (**A**) as well as rabbiteye blueberry leaf extract (**B**) and one internal standard in SRM mode using a Waters Acquity UPLC BEH C18 column. The standards solution mixture was prepared by mixing four phenolic acid and four flavonoid standards, each at a concentration of 1 μg/mL, while the rabbiteye blueberry sample extract was prepared by shaking 0.5 g of sample with 30% ethanol in a 60 °C water bath for 3 h, followed by adopting the same procedure as described in Section 2.3. Peak 1, 5-*O*-caffeoylquinic acid; Peak 2, 3-*O*-caffeoylquinic acid; Peak IS, Epigallocatechin gallate (internal standard); Peak 3, Quercetin-3-*O*-rutinoside; Peak 4, Quercetin-3-*O*-galactoside; Peak 5, Quercetin-3-*O*-glucoside; Peak 6, Kaempferol 3-*O*-glucoside; Peak 7, 3,5-dicaffeoylquinic acid; Peak 8, 4,5-dicaffeoylquinic acid.

By adopting the UPLC-MS/MS separation conditions presented in the Methods section, a total of four phenolic acids and four flavonoids, including 5-*O*-caffeoylquinic acid, 3-*O*-caffeoylquinic acid, quercetin-3-*O*-rutinoside, quercetin-3-*O*-galactoside, quercetin-3-*O*-glucoside, kaempferol-3-*O*-glucoside, 3,5-dicaffeoylquinic acid, and 4,5-dicaffeoylquinic acid, as well as internal standard EGCG, were separated within 7 min and identified by UPLC-MS/MS (Figure 1 and Table 3). Figure S1 shows UPLC-MS/MS chromatograms of phenolic acid and flavonoid standards and rabbiteye blueberry leaf extract, including one internal standard (EGCG), as detected by the SRM mode.

### 3.3. Method Validation and Quantitation

The LOD and LOQ of all eight phenolic acid and flavonoid compounds, as determined by UPLC-MS/MS, ranged from 0.372–0.839 ng/mL and 1.128–2.542 ng/mL, respectively (Table S1). The linear regression equations, along with the coefficient of determinations (R^2^) obtained from the calibration curves of eight phenolic acid and flavonoid compounds, are also shown in Table S1. Table 3 also shows the contents of eight phenolic acid and flavonoid compounds, with their amounts ranging from 60.8–6474.2 μg/g. Obviously, 3-*O*-caffeoylquinic acid, quercetin-3-*O*-galactoside, and quercetin-3-*O*-rutinoside dominated in the NTU 104 cultivar of the rabbiteye blueberry leaves used in our study. Similar outcomes were reported by Cezarotto et al. [36] and Wu et al. [35]. 

Both the repeatability (intra-day variability) and intermediate precision (inter-day variability) data are shown in Table S2, with the relative standard deviation (RSD) being from 1.14–4.83% for the former and from 1.61–6.33% for the latter. All the precision data are in agreement with a report issued by TFDA [37], stating that the RSD should be <10% for the analyte concentration ≥1 ppm for both repeatability and intermediate precision data. Similarly, the recovery data of phenolic acid and flavonoid compounds in rabbiteye blueberry leaves ranged from 90.37–102.65% (Table S3), which also meets the requirement set by TFDA [37], stating that the recovery should be from 85–110% for the analyte concentration ≥100 ppm. Thus, all the method validation data demonstrated that a high precision and accuracy was achieved for the method employed in this study. 

### 3.4. Characteristics of Rabbiteye Blueberry Nanoemulsion

**Figure 2:**
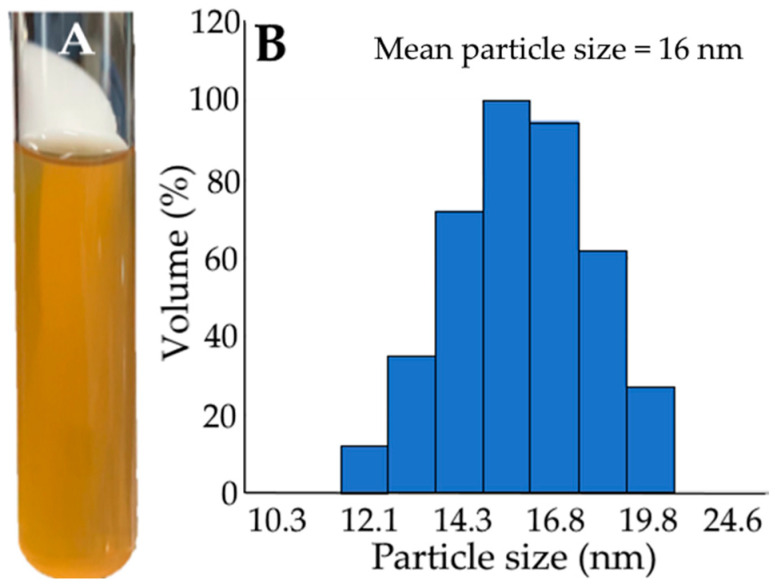

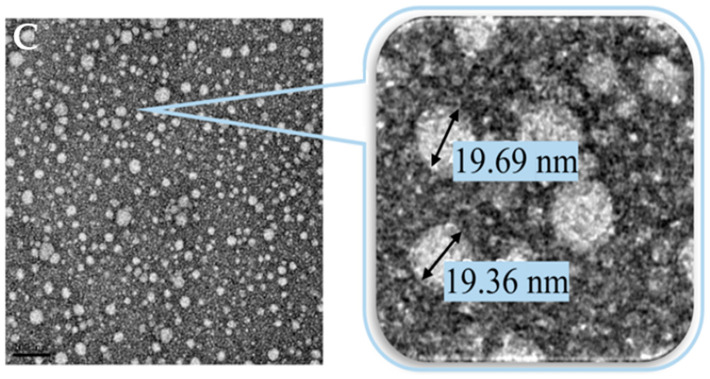
Appearance (**A**), particle size distribution as determined by DLS (**B**), and TEM image (**C**) of rabbiteye blueberry leaf nanoemulsion.

Figure 2 shows the appearance of blueberry nanoemulsion (2A), particle size distribution (2B), and TEM images (2C). As mentioned in the Methods section, a 10 mL transparent nanoemulsion containing four phenolic acids (5 mg/mL), including 3-*O*-caffeoylquinic acid, 5-*O*-caffeoylquinic acid, 3,5-dicaffeoylquinic acid, and 4,5-dicaffeoylquinic acid, was successfully prepared, with the mean particle size being 16.0 nm as determined by DLS and 19 nm by TEM, as well as PDI being 0.214 and zeta-potential −53.8 mV. Both DLS and TEM analyses showed a similar mean particle size of the blueberry leaf nanoemulsion. Additionally, the encapsulation efficiency based on the content of four phenolic acids was calculated to be 84.13%, which should be responsible for the nanoemulsion’s high stability during storage at 4℃ for 3 months (Table 4) and during heating at 40–100 °C for 2 h (Table 5), as only a slight change in mean particle size, PDI, and zeta potential was observed. However, we must point out here that the total amount of four phenolic acids was used as the indicator components instead of flavonoids for calculation of encapsulation efficiency, as the former was shown to be present at a much higher level in rabbiteye blueberry leaves. Similarly, a high stability was found for the catechin nanoemulsion prepared from green tea leaf waste [38] and from avocado peel [39].

### 3.5. Animal Experiment

In this study we injected D-galactose into mice to induce aging, as it has been established that the intake of D-galactose in excess can cause metabolic abnormality of non-enzymatic glycation and results in the production of a large amount of reactive oxygen species (ROS), leading to the impairment of normal cells for subsequent aging [40,41]. The effect of administration of rabbiteye blueberry leaf extract and nanoemulsion on body weight in D-galactos-induced aged mice is shown in Table 6. Following 12-week administration, the body weight of the induction group (D) declined by 3.82% compared to the control group (C). However, compared to the induction group (D), the body weight was higher by 1.66%, 1.32%, 8.28%, and 4.64% for the LE, HE, LN, and HN groups, respectively. Nevertheless, the difference in body weight among the LE, HE, LN, and HN groups remained insignificant (*p* > 0.05). This outcome implied that the injection of D-galactose had a mild impact on the body weight of mice for all six groups. A similar result was reported by Tsai and Yin [42], who studied the anti-glycative and anti-inflammatory effects of protocatechuic acid on brain of mice treated by D-galactose.

**Figure 3:**
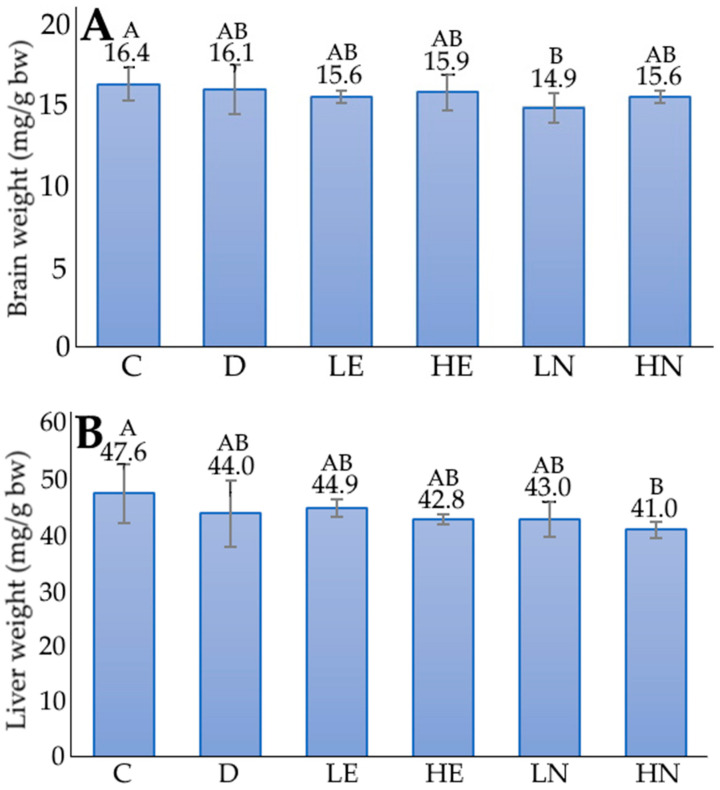
Effect of administration of blueberry leaf extract and nanoemulsion on brain weight (**A**) and liver weight (**B**) in D-galactose-induced aged mice. C, control group; D, induction group; LE, low-dose extract group; HE, high-dose extract group; LN, low-dose nanoemulsion group; HN, high-dose nanoemulsion group. Data are presented as means ± standard deviation (*n* = 6) and the data with different capital letters (A,B) are significantly different at *p* < 0.05.

Figure 3 shows the effect of administration of blueberry leaf extract and nanoemulsion on brain weight and liver weight in D-galactose-induced aged mice. Following 12-week administration, only a slight change in brain weight and liver weight was shown for all the treatments. This finding indicated that the D-galactose injection only had a mild impact on both brain and liver weights in mice during administration for 12 weeks.

**Figure 4:**
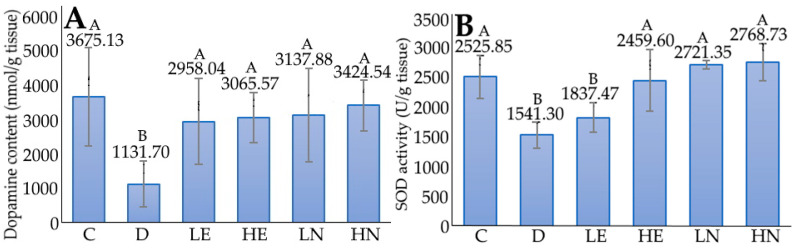

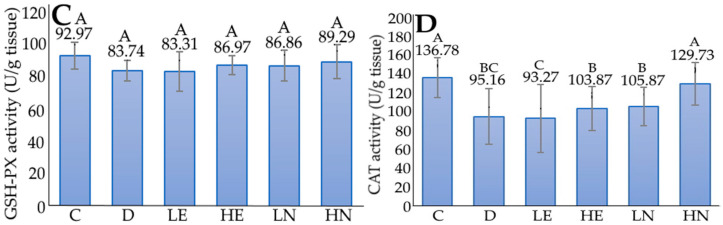
The effect of administration of the blueberry leaf extract and nanoemulsion on dopamine content (**A**) in brain as well as SOD (**B**), GSH-Px (**C**), and CAT (**D**) activities in the liver of D-galactose-induced aged mice. C, control group; D, induction group; LE, low-dose extract group; HE, high-dose extract group; LN, low-dose nanoemulsion group; HN, high-dose nanoemulsion group. Data are presented as means ± standard deviation (*n* = 6) and the data with different capital letters (A–C) are significantly different at *p <* 0.05.

The effect of administration of blueberry leaf extract and nanoemulsion on the brain dopamine content in D-galactose-induced aged mice is shown in Figure 4. Compared to the induction group (D), the brain dopamine content of the control group (C) rose by 224.74%. Likewise, for the other groups, the brain dopamine contents increased pronouncedly by 161.38%, 170.88%, 177.27%, and 202.60% for the LE, HE, LN, and HN groups, respectively, with the HN group showing the most pronounced rise. It may be postulated that the blueberry leaf nanoemulsion at high dose was capable of crossing the blood–brain barrier for the activation of substantia nigra cells to secrete more dopamine. In several previous studies, Krzysztoforska et al. [43] reported that the dopamine contents were raised by 115%, 96%, and 596% in the striatum, hippocampus, and prefrontal cortex, respectively, following the administration of protocatechuic acid at 100 mg/kg to D-galactose-induced mice for 48 days. Likewise, Wang et al. [44] found an elevation in dopamine level by 13% and 17% following the administration of 50 and 300 mg/kg of enzyme degradation extract from *Porphyra yezoensis* in their study dealing with alleviation of oxidative stress and brain injury in D-galactose-induced aging mice. In addition, the anthocyanin extract prepared from black chokeberry was shown to raise dopamine contents by 32% and 47%, respectively, following administration at a dose of 15 and 30 mg/kg to D-galactose-induced mice for eight weeks. By comparison, the brain dopamine contents in D-galactose-induced mice in our study were shown to raise to a much higher level, which should be due to a higher capability of the blueberry leaf nanoemulsion crossing the blood–brain barrier as mentioned above when compared to the extract.

Liver is a vital organ in the human body. However, it can be susceptible to attacks by ROS. It was reported that both sinusoidal endothelial cells and bile duct cells in the liver are highly sensitive to the impairment caused by oxidative stress, and the apoptosis of these cells can lead to liver aging [45]. Thus, the presence of enzymatic and non-enzymatic antioxidants plays a vital role in maintaining normal function of liver. Figure 4 shows the effect of administration of blueberry leaf extract and nanoemulsion on superoxide dismutase (SOD) activity in the liver of D-galactose-induced aged mice. Compared to the control group (C), the SOD activity of the induction group (D) was significantly lower (*p* < 0.05) by 38.98%. However, the SOD activity of the HE, LN, and HN groups were significantly higher (*p* < 0.05) than the D group by 59.58%, 76.56%, and 79.64%, respectively, while no significant difference (*p* > 0.05) in SOD activity was found between the LE and D groups. A similar trend was shown for the glutathione peroxidase (GSH-Px) activity, as evident by a rise of 3.86%, 3.73%, and 6.63% for the HE, LN, and HN groups, respectively, when compared to the D group (Figure 4). Interestingly, for catalase (CAT) activity, an increase of 9.16%, 11.26%, and 36.33% was shown for the HE, LN, and HN groups, respectively, when compared to the D group (Figure 4). However, no significant difference (*p* > 0.05) in CAT activity was observed among the HE, LN, and D groups. Additionally, the CAT activity between the HN and C group was insignificant (*p* > 0.05). These outcomes clearly revealed that the antioxidant activities in mice liver were elevated substantially following the administration of blueberry leaf nanoemulsion at a high dose.

**Figure 5:**
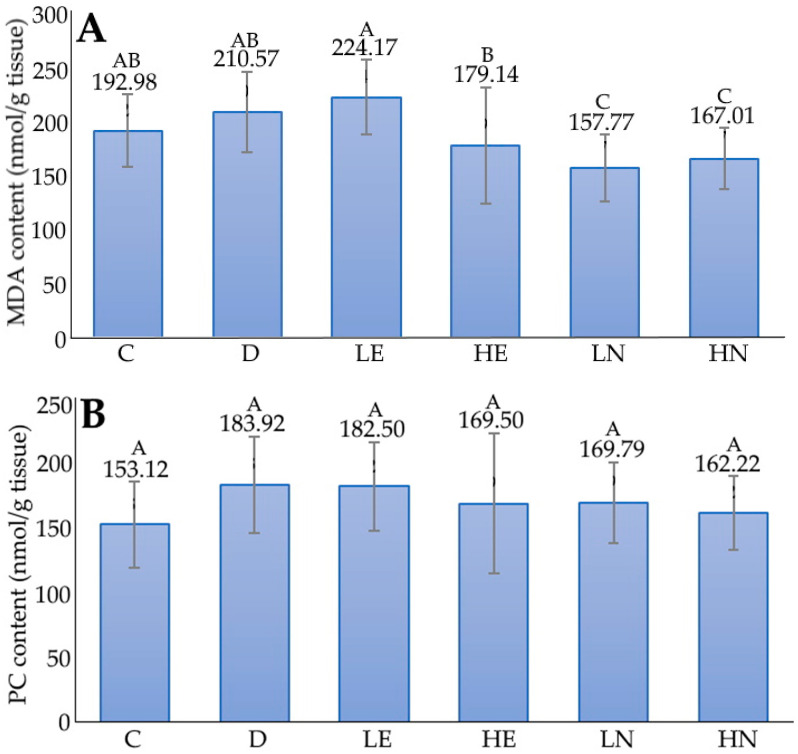
The effect of administration of blueberry leaf extract and nanoemulsion on malondialdehyde (MDA) (**A**) and protein carbonyl (PC) (**B**) contents in the brains of D-galactose-induced aged mice. C, control group; D, induction group; LE, low-dose extract group; HE, high-dose extract group; LN, low-dose nanoemulsion group; HN, high-dose nanoemulsion group. Data are presented as means ± standard deviation (*n* = 6) and the data with different capital letters (A–C) are significantly different at *p <* 0.05.

The effect of administration of blueberry leaf extract and nanoemulsion on malondialdehyde (MDA) content in the brain of D-galactose-induced aged mice is shown in Figure 5. Compared to the C group, the MDA content of the D group was significantly higher (*p* < 0.05) by 9.11%. However, compared to the D group, the MDA contents of the HE, LN and HN groups were significantly lower (*p* < 0.05) by 14.93%, 25.07%, and 20.69%, respectively. Like MDA, a similar outcome was observed for the protein carbonyl (PC) content in the brains of D-galactose-induced aged mice (Figure 5). A decline in PC content of 0.77%, 7.84%, 7.69%, and 11.80% was shown for the groups of LE, HE, LN, and HN groups, respectively, when compared to the D group. However, the difference among these groups was insignificant (*p* > 0.05). As both MDA and PC are vital indexes of lipid peroxidation, the results obtained in our study indicated that both blueberry leaf extract and nanoemulsion may be effective in decreasing oxidative stress, with the nanoemulsion at a high dose showing the most pronounced effect.

The elevation of antioxidant enzyme activity and decrease of MDA and PC contents in liver of D-galactose-induced aged mice following administration of polyphenol-rich extracts have been well documented. For instance, Guo et al. [46] observed a raise in both SOD and GSH-Px activities in the liver by 232% and 109%, as well as a decline in MDA content in the brain by 88% following a 10-week administration of 100 mg/kg of polyphenol-rich *Apocynum venetum* extract in D-galactose-induced oxidative stress in mice. Similarly, a dose-dependent decrease was shown for the PC content in the brain of D-galactose-induced aged mice, which was reduced by 51% following the administration of protocatechuic acid at 2 mg/kg for 8 weeks [42]. In a study dealing with the anti-aging and redox state regulation effects of A-type proanthocyanidins-rich cranberry concentration and its comparison with grape seed extract in mice, Jiao et al. [47] reported that, following administration of the former and the latter at 30 mg/kg for 8 weeks, the MDA contents in mice brain were respectively reduced by 25% and 30%, while the TBARS level in mice liver were diminished by 30–56% and 35–57% at low (15 mg/kg), moderate (30 mg/kg), and high (60 mg/kg) doses.

Furthermore, the hepatic GSH-Px activity in mice was enhanced by 42% for the cranberry concentrate treatment at 60 mg/kg, while the SOD activity was raised by 13% for the grape seed extract at 30 mg/kg. Surprisingly, for the cranberry concentrate treatment at 15, 30, and 60 mg/kg, the CAT activities decreased by 40%, 31%, and 33% in mice, respectively [47]. This result indicated that the antioxidant enzyme activities may be varied depending on sample variety and preparation method. In another report, the activities of SOD, GSH-Px, and CAT in D-galactose-induced mice liver were shown to increase by 30%, 63%, and 28%, respectively, following the administration of the ethanol extract from *Ficus vasculosa* at 50 mg/kg for 8 weeks, which can be due to the presence of polyphenols in the ethanol extract [48]. Moreover, a higher dose at 200 mg/kg was found to result in a rise of the SOD, GSH-Px, and CAT activities by 49%, 76%, and 38%, respectively, demonstrating a dose-dependent increase in the antioxidant enzyme activities of D-galactose-induced mice liver.

In another study, Yu et al. [49] compared the antioxidant capacity and phenolic compounds of oat and buckwheat vinegar during the production process. The activities of SOD, GSH-Px, and CAT in D-galactose-induced mice liver were elevated by 29%, 29%, and 24%, respectively, when compared to the induction group, following administration of the oat vinegar at 5 mg/kg for 8 weeks, while for buckwheat vinegar at the same dose, the antioxidant enzyme activities were raised by 29%, 30%, and 26%, respectively. This outcome implied that both oat and buckwheat vinegars showed a similar antioxidant enzyme activity in D-galactose-induced mice liver, which can be attributed to the presence of phenolic compounds in both vinegars. Taken together, all the results showed above demonstrated that the presence of phenolic compounds in plants extracts play a vital role in decreasing oxidative stress in D-galactose-induced mice liver through enhancement of the antioxidant enzyme activities.

Theoretically, senescence can result in a decline of dopamine, norepinephrine, and serotonin in the frontal sinus of brain for a subsequent decrease of volume and function in the prefrontal cortex of brain. Furthermore, the behavior disorder expressed by the elderly is similar to that expressed by patients with frontal lobe lesion, which in turn failed to inhibit interference information, leading to a persistent error for a drop in capability of working memory [50]. Thus, it can be inferred that the defect of prefrontal lobe is the main reason responsible for behavior disorder. Additionally, the white matter of the frontal lobe of brain degenerates faster following a rise in age, which may affect the memory circuit of the prefrontal cortex. Research has also shown that the dopamine loss is partially responsible for the memory loss of patients with Alzheimer’s disease [50]. More specifically, like other monoamine neurotransmitters, dopamine is a brain secretion and shows its function for regulation of rapid neurotransmission-mediated glutamic acid and r-aminobutyric acid (GABA). Additionally, dopamine shows a vital physiological function in olfactory modulation, immune system, sympathetic nervous system, cardiovascular, retina, kidney, and hormone regulation [51,52,53,54,55,56]. However, the most notable disease which can be closely associated with dopamine is Parkinson’s disease, which can be due to the inadequate secretion of dopamine caused by apoptosis of substantia nigra cells [57]. The maladjustment of dopamine can lead to attention deficit hyperactivity disorder, depression, and Tourette’s syndrome [58,59,60]. Huntington’s disease, caused by the degeneration of the brain striatum, also plays a vital role in the pathogenesis of Parkinson’s disease, as the striatum is a brain region rich in dopamine for signal transduction.

Taken together, a total of four phenolic acids and four flavonoids were extracted, separated, identified, and quantified in Rabbiteye blueberry leaves by UPLC-MS/MS in SRM mode, followed by the preparation of extract and nanoemulsion to evaluate the anti-aging activity in mice, with the latter showing a more marked effect. Of the two nanoemulsion doses (8 and 40 mg/kg bw) tested, the nanoemulsion at a high dose (40 mg/kg bw) was shown to be more effective in alleviating the D-galactose-induced aging in mice brain by elevating the dopamine level and reducing the levels of malondialdehyde and protein carbonyl, as well as increasing the activities of antioxidant enzymes, including superoxide dismutase, glutathione peroxidase, and catalase in mice liver. The observed anti-aging effect by rabbiteye blueberry leaf nanoemulsion in mice could be attributed to the facilitated uptake of nanoemulsion particles by cells. As cell membranes are mostly negatively charged, a strong binding and eventual uptake of nanoemulsion particles by cells is feasible only if the particles are positively charged [61,62]. In other words, the positively-charged nanoemulsion particles have a strong binding for efficient absorption by cells and higher bioactivity when compared to the negatively-charged and neutral ones [62]. However, the rabbiteye blueberry leaf nanoemulsion prepared in this study with a negative surface charge (zeta potential, −54 mV) has shown a pronounced anti-aging effect in mice, implying that the uptake of nanoemulsion particles by cells was not caused by electrostatic binding, but may be due to some other types of interactions. Several comprehensive review articles have highlighted that the cellular uptake of negatively-charged or neutral nanoparticles is possible through strong and non-specific interactions with the plasma membrane, followed by one or a combination of five endocytosis mechanisms including phagocytosis, clathrin-mediated endocytosis, caveolin-mediated endocytosis, clathrin/caveolae-independent endocytosis, and micropinocytosis [62,63]. Although these five different endocytosis mechanisms are the major uptake processes for nanoparticles to enter into cells, some other uptake mechanisms have also been reported, including hole formation, passive diffusion, electroporation, and direct microinjection [62]. Moreover, several studies have demonstrated the internalization of negatively-charged tanshinone nanoemulsion and lycopene-nanogold nanoemulsion particles into lung cancer cells (A549) and colon cancer cells (HT-29), respectively [64,65]. Overall, the antiaging effects demonstrated in this study may be caused by the cellular uptake of negatively-charged rabbiteye blueberry leaf nanoemulsion through non-specific interactions and subsequent endocytosis.

## 4. Conclusions

In conclusion, an UPLC-MS/MS method was developed to determine four phenolic acids and four flavonoids in rabbiteye blueberry leaves with 3-*O*-caffeoylquinic acid present in the highest amount, followed by quercetin-3-*O*-galactoside, quercetin-3-*O*-rutinoside, quercetin-3-*O*-glucoside, 5-*O*-caffeoylquinic acid, kaempferol-3-*O*-glucoside, 3,5-dicaffeoylquinic acid, and 4,5-dicaffeoylquinic acid. The blueberry leaf nanoemulsion was prepared by using an appropriate ratio of blueberry leaf extract, soybean oil, Tween 80, ethanol, glycerol, and deionized water, with the average particle size and zeta potential being 16 nm and −54 mV, respectively. Comparatively, the high-dose nanoemulsion was the most efficient in elevating the dopamine content in mice brains, as well as the activities of SOD, GPH-Px, and CAT in mice liver, while reducing the contents of MDA and PC in mice brain. Taken together, the high-dose rabbiteye blueberry leaf nanoemulsion possesses great potential to be developed into a health food.

## Figures and Tables

**Table 1:** Effect of different ethanol proportions on total phenolic content (TPC) (mg/g) and total flavonoid content (TFC) (mg/g) in rabbiteye blueberry leaves ^a^.

	30% EtOH	50% EtOH	70% EtOH
TPC ^b^	132.80 ± 3.92 ^A^	136.19 ± 4.04 ^A^	116.39 ± 6.27 ^B^
TFC ^c^	206.19 ± 5.51 ^A^	194.66 ± 1.34 ^B^	164.19 ± 6.48 ^B^

^a^ Data are presented as mean ± standard deviation (*n* = 3) and data bearing different capital letters (A,B) in the same row are significantly different (*p* < 0.05). ^b^ Expressed as gallic acid equivalent. ^c^ Expressed as quercetin equivalent.

**Table 2:** The total phenolic acid content (TPC) (mg/g) and total flavonoid content (TFC) (mg/g) in different cultivars of rabbiteye blueberry leaves ^a,b^.

Species	TPC ^c^	TFC ^d^	Species	TPC ^b^	TFC ^c^
1. Gulf coast1	32.43 ± 0.05 ^A,l^	13.12 ± 2.61 ^B,k^	11. GGM	82.70 ± 1.77 ^A,f^	22.72 ± 0.66 ^B,h^
2. Sharp blue	66.31 ± 4.69 ^A,h^	51.25 ± 1.57 ^B,b^	12. Tifblue	119.52 ± 1.04 ^A,b^	37.83 ± 0.63 ^B,d^
3. Climax	47.30 ± 0.52 ^A,j^	15.27 ± 0.38 ^B,j^	13. Sunshine Blue	30.01 ± 1.04 ^A,l^	18.34 ± 0.06 ^B,i^
4. Misty	104.16 ± 2.88 ^B,c^	111.36 ± 2.67 ^A,a^	14. NTU 042	22.57 ± 0.94 ^A,m^	9.12 ± 0.34 ^B,l^
5. O’Neal	59.16 ± 6.47 ^A,i^	32.27 ± 1.29 ^B,e^	15. NTU 03	32.12 ± 0.49 ^A,l^	15.16 ± 0.03 ^B,j^
6. Gloria	88.51 ± 9.95 ^A,e^	21.05 ± 1.51 ^B,h^	16. Gulf coast2	38.05 ± 0.71 ^A^.^k^	30.20 ± 0.50 ^B,f^
7. Fuku	77.28 ± 4.00 ^A,fg^	29.45 ± 0.44 ^B,f^	17. Onel	64.37 ± 2.21 ^A,hi^	37.72 ± 0.63 ^B,d^
8. Powderblue	93.02 ± 0.49 ^A,de^	26.95 ± 0.96 ^B,g^	18. Sunshine	21.57 ± 0.85 ^A,m^	14.47 ± 0.44 ^B,jk^
9. Gwl coast	51.30 ± 1.51 ^A,j^	43.69 ± 1.68 ^B,c^	19. NTU 104 (fresh leaves)	136.09 ± 2.72 ^A,a^	36.25 ± 1.07 ^B,d^
10. Alice	73.66 ± 0.71 ^A,g^	21.83 ± 0.16 ^B,h^	20. NTU 104 (withered leaves)	97.20 ± 3.75 ^A,d^	29.09 ± 0.38 ^B,f^

^a^ Data are presented as mean ± standard deviation (*n* = 3). ^b^ Data with different capital letters (A–B) in the same row represent significantly different TPC and TFC for the same rabbiteye blueberry species, while different small letters (a–m) in the same column represent significantly different TPC or TFC for different rabbiteye blueberry species (*p* < 0.05). ^c^ Expressed as gallic acid equivalent. ^d^ Expressed as quercetin equivalent.

**Table 3:** Identification and quantitation data of phenolic acids and flavonoids in rabbiteye blueberry leaf extract by UPLC-MS/MS.

Peak No.	Compound	Retention Time (min)		MS/MS (*m*/*z*) ^b^		Content (µg/g) ^c^
		Standard	Sample	Precursor Ion	Product Ion	
1	5-*O*-caffeoylquinic acid ^a^	2.92	2.79	353	191, 179	815.8 ± 18.7
2	3-*O*-caffeoylquinic acid ^a^	3.18	3.07	353	191, 173	6474.2 ± 105.58
3	quercetin-3-*O*-rutinoside ^a^	4.08	3.98	609	271, 300	1036.6 ± 17.2
4	quercetin-3-*O*-galactoside ^a^	4.38	4.26	463	300	1943.9 ± 125.8
5	quercetin-3-*O*-glucoside ^a^	4.54	4.46	463	301, 271	867.2 ± 39.2
6	kaempferol 3-*O*-glucoside ^a^	5.48	5.38	447	284, 255	309.7 ± 15.4
7	3,5-dicaffeoylquinic acid ^a^	5.54	5.48	515	173, 353	195.3 ± 13.1
8	4,5-dicaffeoylquinic acid ^a^	6.41	6.24	515	191, 353	60.8 ± 3.3

^a^ Peaks were positively identified based on comparison of retention time and mass spectra of unknown peaks in rabbiteye blueberry leaf extract with that of standards. ^b^ The MS/MS data (precursor ion and product ion) obtained for eight phenolic acid and flavonoid compounds in both standard solution and rabbiteye blueberry leaf extract were found to be the same. ^c^ Data are presented as mean ± standard deviation (*n* = 3).

**Table 4:** Particle size, polydispersity index and zeta-potential of rabbiteye blueberry leaf nanoemulsion during storage for 90 days at 4 °C.

Day	Particle Size (nm) ^a^	Polydispersity Index ^b^	Zeta-Potential (mV) ^b^
0	16.0	0.214 ± 0.011 ^A^	−53.8 ± 1.9 ^A^
7	16.2	0.230 ± 0.013 ^B^	−52.2 ± 0.9 ^A^
21	15.4	0.235 ± 0.007 ^B^	−49.9 ± 0.9 ^B^
30	15.7	0.249 ± 0.003 ^B^	−43.2 ± 1.1 ^C^
60	15.8	0.246 ± 0.013 ^B^	−41.6 ± 1.2 ^C^
90	15.0	0.249 ± 0.007 ^B^	−39.2 ± 1.3 ^D^

^a^ Data are presented as the mean of triplicate analyses. ^b^ Data are presented as the mean ± standard deviation (*n* = 3) and data with different capital letters (A–D) in the same column are significantly different at *p* < 0.05.

**Table 5:** Particle size, polydispersity index and zeta-potential of rabbiteye blueberry leaf nanoemulsion during heating at 40–100 °C for varied time length.

Temp	Particle Size (nm) ^a^					Polydispersity Index ^b^					Zeta-Potential (mV) ^b^				
	0 h	0.5 h	1 h	1.5 h	2 h	0 h	0.5 h	1 h	1.5 h	2 h	0 h	0.5 h	1 h	1.5 h	2 h
40 °C	16.0 ^A^	12.5 ^BC^	14.3 ^A^	12.8 ^A^	14.2 ^A^	0.214	0.223	0.237	0.258	0.263	−53.8 ^A^	−46.9 ^A^	−42.4 ^A^	−40.5 ^A^	−39.7 ^A^
60 °C	16.0 ^A^	11.6 ^C^	15.0 ^A^	12.6 ^A^	12.8 ^C^	0.214	0.235	0.241	0.249	0.271	−53.8 ^A^	−43.2 ^B^	−38.9 ^B^	−36.2 ^B^	−34.0 ^B^
80 °C	16.0 ^A^	15.2 ^A^	12.2 ^B^	11.6 ^B^	13.4 ^B^	0.214	0.229	0.228	0.251	0.259	−53.8 ^A^	−39.6 ^C^	−39.6 ^B^	−38.5 ^A^	−31.4 ^C^
100 °C	16.0 ^A^	13.1 ^B^	11.8 ^B^	11.3 ^B^	11.2 ^D^	0.214	0.232	0.245	0.243	0.258	−53.8 ^A^	−37.9 ^D^	−36.6 ^C^	−35.4 ^B^	−31.1 ^C^

^a^ Data are shown as the mean of triplicate analysis. ^b^ Data are shown as mean ± standard deviation (*n* = 3) and data with different capital letters (A–D) in the same column are significantly different at *p* < 0.05.

**Table 6:** The effect of the administration of rabbiteye blueberry leaf extract and nanoemulsion on body weight in D-galactose-induced aged mice.

Group	Body Weight (g)					
	Week 1	Week 2	Week 3	Week 4	Week 5	Week 6
C	26.0 ± 2.8 ^A^	26.7 ± 2.4 ^B^	27.5 ± 2.0 ^AB^	28.2 ± 1.9 ^A^	28.2 ± 2.0 ^A^	29.0 ± 2.1 ^A^
D	26.4 ± 1.4 ^A^	27.4 ± 1.7 ^AB^	27.6 ± 1.4 ^AB^	27.9 ± 1.6 ^A^	28.3 ± 1.7 ^A^	28.7 ± 1.7 ^A^
HE	26.5 ± 1.0 ^A^	27.6 ± 1.3 ^AB^	27.3 ± 0.8 ^B^	27.9 ± 0.8 ^A^	28.1 ± 1.2 ^A^	29.0 ± 1.2 ^A^
HN	27.2 ± 0.9 ^A^	28.1 ± 0.6 ^AB^	28.4 ± 0.7 ^AB^	28.8 ± 0.8 ^A^	28.8 ± 0.8 ^A^	29.5 ±1.0 ^A^
LE	26.6 ± 0.8 ^A^	27.5 ± 0.8 ^AB^	28.1 ± 0.6 ^AB^	28.5 ± 0.7 ^A^	28.4 ± 0.6 ^A^	29.2 ± 0.7 ^A^
LN	28.1 ± 1.6 ^A^	29.0 ± 1.7 ^A^	29.3 ± 1.6 ^A^	29.4 ± 1.6 ^A^	29.8 ± 1.9 ^A^	30.5 ± 1.9 ^A^
	**Week 7**	**Week 8**	**Week 9**	**Week 10**	**Week 11**	**Week 12**
C	28.6 ± 2.1 ^A^	29.6 ± 2.0 ^AB^	29.4 ± 1.6 ^A^	29.8 ± 1.8 ^AB^	29.9 ±1.7 ^AB^	31.4 ± 1.8 ^AB^
D	28.7 ± 1.6 ^A^	28.9 ± 1.4 ^A^	28.9 ± 1.4 ^A^	28.9 ± 1.7 ^B^	28.7 ± 1.7 ^B^	30.2 ± 1.7 ^B^
HE	28.7 ± 1.3 ^A^	29.6 ± 0.9 ^AB^	29.1 ± 1.3 ^A^	29.4 ± 1.3 ^B^	29.6 ± 1.3 ^AB^	30.6 ± 1.4 ^B^
HN	29.3 ± 1.0 ^A^	30.3 ± 0.8 ^AB^	29.8 ± 1.1 ^A^	30.3 ± 1.0 ^AB^	30.4 ± 0.9 ^AB^	31.6 ± 0.7 ^AB^
LE	28.8 ± 0.6 ^A^	30.1 ± 0.9 ^AB^	30.1 ± 0.7 ^A^	30.3 ± 0.9 ^AB^	30.1 ± 0.8 ^AB^	30.7 ± 1.0 ^AB^
LN	30.1 ± 2.0 ^A^	31.2 ± 2.3 ^B^	30.9 ± 2.1 ^A^	31.6 ± 2.0 ^A^	31.2 ± 1.9 ^A^	32.7 ± 1.9 ^A^

Data shown are mean ± standard deviation (*n* = 6) and the data with different capital letters (A,B) in the same column are significantly different at *p* < 0.05. C, control group; D, induction group; LE, low-dose extract group; HE, high-dose extract group; LN, low-dose nanoemulsion group; HN, high-dose nanoemulsion group.

## Data Availability

Data is contained within the article or supplementary material.

## Supplementary Materials

The following supporting information can be downloaded at: https://www.mdpi.com/article/10.3390/foods12101942/s1, Figure S1: UPLC-MS/MS chromatograms of phenolic acid and flavonoid standards (A) and rabbiteye blueberry leaf extract (B) as well as internal standard EGCG as detected by SRM mode; Table S1: Linear regression equation and coefficient of determination of phenolic acid and flavonoid compounds in rabbiteye blueberry leaves as determined by UPLC-MS/MS; Table S2: Quality control data of phenolic acid and flavonoid compounds in rabbiteye blueberry leaves as determined by UPLC-MS/MS; Table S3: Recovery of phenolic acid and flavonoid compounds in rabbiteye blueberry leaves by UPLC-MS/MS.
