# Supplementary material for: Analysis of Phenolic Acids and Flavonoids in Rabbiteye Blueberry Leaves by UPLC-MS/MS and Preparation of Nanoemulsions and Extracts for Improving Antiaging Effects in Mice
Source: Foods. 2023 May 10;12(10):1942. doi: 10.3390/foods12101942 (PMC10216987; doi:10.3390/foods12101942)
Supplement: Supplementary file 1 [file foods-12-01942-s001.zip › foods-2366876-supplementary.pdf]

Supplementary Material

for

Analysis of Phenolic Acids and Flavonoids in Rabbiteye Blueberry Leaves by UPLC-MS/MS and Preparation of Nanoemulsions and Extracts for Improving Antiaging Effects in Mice

Hsin-Rong Yu[1] and Bing-Huei Chen[1,2,*]

[1] Department of Food Science, Fu Jen Catholic University, New Taipei City 242062, Taiwan
[2] Department of Nutrition, China Medical University, Taichung 40402, Taiwan
* Correspondence: 002622@mail.fju.edu.tw, Telephone: 886-2-29053626.

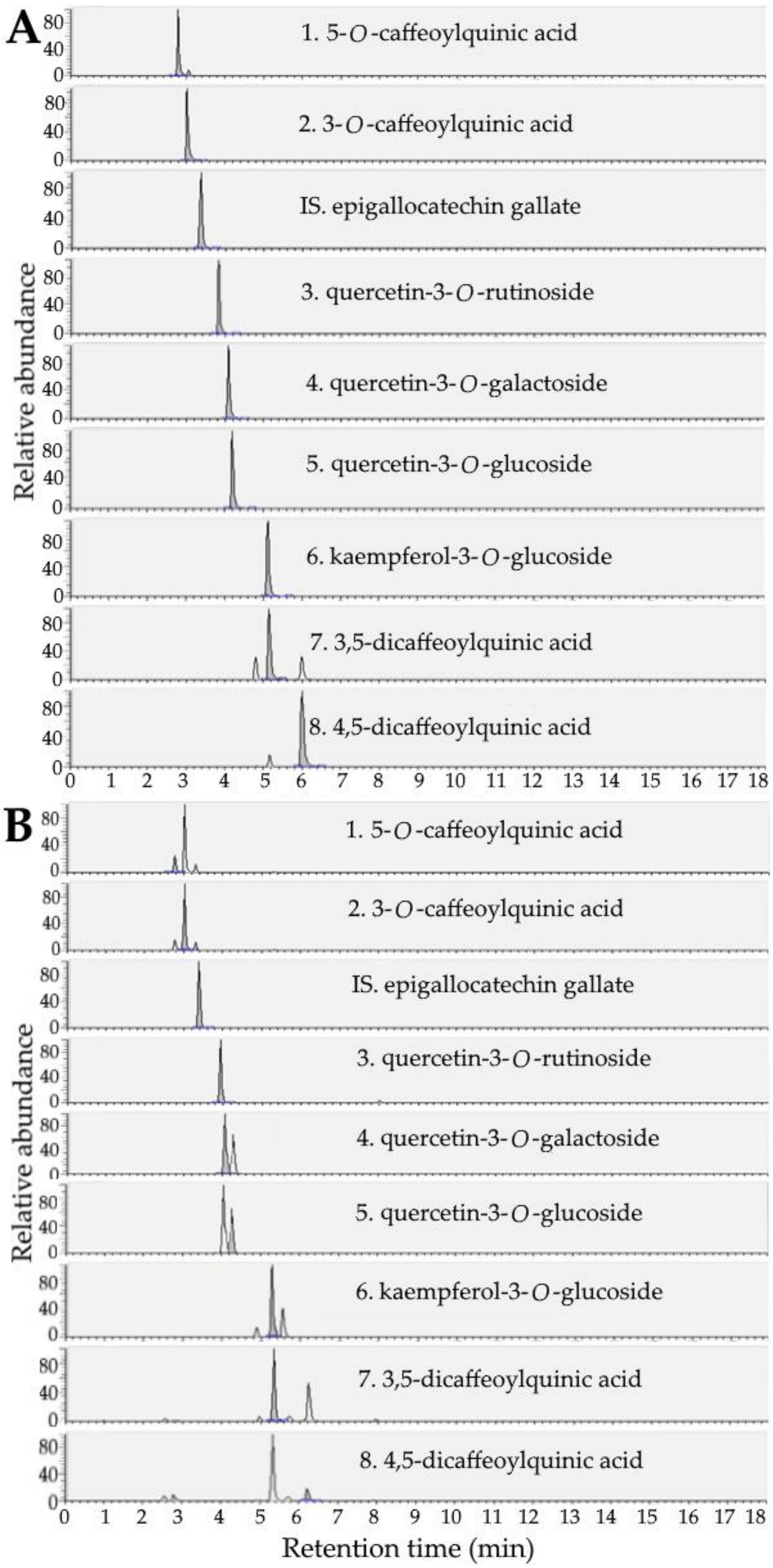

Figure S1. UPLC-MS/MS chromatograms of phenolic acid and flavonoid standards (A) and rabbiteye blueberry leaf extract (B) as well as internal standard EGCG as detected by SRM mode.

Table S1. Linear regression equation and coefficient of determination of phenolic acid and flavonoid compounds in rabbiteye blueberry leaves as determined by UPLC-MS/MS.

Compounds	LOD (ng/mL)[a]	LOQ (ng/mL)[b]	Linear regression equation	R^2 [c]
3-*O*-caffeoylquinic acid	0.645	1.954	y=2500957x+2260	0.9901
5-*O*-caffeoylquinic acid	0.431	1.306	y=4387038x-508	0.9955
3,5-dicaffeoylquinic acid	0.373	1.130	y=3398226x+48	0.9967
4,5-dicaffeoylquinic acid	0.430	1.304	y=4895063x-384	0.9956
quercetin-3-*O*-galactoside	0.773	2.344	y=10856394x+744	0.9858
quercetin-3-*O*-glucoside	0.839	2.542	y=7523880x-1507	0.9833
quercetin-3-*O*-rutinoside	0.440	1.333	y=10931313x-1107	0.9954
kaempferol-3-*O*-glucoside	0.372	1.128	y=12192000x-2938	0.9967

[a]Limit of detection

[b]Limit of quantitation

[c]Coefficient of determination

Table S2. Quality control data of phenolic acid and flavonoid compounds in rabbiteye blueberry leaves as determined by UPLC-MS/MS.

Compounds	Intra-day variability[a]		Inter-day variability[a]	
	Mean ± SD (μg/g)	RSD (%)[b]	Mean ± SD (μg/g)	RSD (%)[b]
5-*O*-caffeoylquinic acid	822.59 ± 25.48	3.10	815.83 ± 18.75	2.30
3-*O*-caffeoylquinic acid	6519.64 ± 74.01	1.14	6479.53 ± 117.99	1.82
quercetin-3-*O*-rutinoside	1044.11 ± 14.06	1.35	1038.45 ± 16.70	1.61
quercetin-3-*O*-galactoside	2001.05 ± 86.88	4.34	1950.76 ± 123.43	6.33
quercetin-3-*O*-glucoside	805.12 ± 20.90	2.60	910.42 ± 20.48	2.25
kaempferol-3-*O*-glucoside	324.59 ± 15.68	4.83	322.78 ± 12.63	3.91
3,5-dicaffeoylquinic acid	202.45 ± 9.59	4.73	194.50 ± 9.36	4.81
4,5-dicaffeoylquinic acid	66.09 ± 2.58	3.90	61.15 ± 2.99	4.88

[a]Mean of triplicate analyses ± standard deviation.

[b]Relative standard deviation (%) = (standard deviation/mean) × 100.

Table S3. Recovery of phenolic acid and flavonoid compounds in rabbiteye blueberry leaves by UPLC-MS/MS.

Compound	Original (μg)[a]	Spiked (μg)[b]	Found (μg)[c]	Recovery (%)[d]	Mean ± SD (%)	RSD (%)[e]
3-*O*-caffeoylquinic acid	3180.22	350	3615.30	102.41	102.61 ± 6.20	6.05
		3500	6867.93	102.81		
5-*O*-caffeoylquinic acid	444.86	45	453.02	92.48	92.92 ± 4.77	5.13
		450	836.16	93.44		
3,5-dicaffeoylquinic acid	104.74	10.5	108.44	94.10	95.01 ± 6.26	6.59
		105	201.20	95.93		
4,5-dicaffeoylquinic acid	33.37	3.5	35.80	97.10	95.80 ± 6.83	7.13
		35	64.62	94.51		
quercetin-3-*O*-galactoside	1019.57	110	1107.20	98.02	102.65 ± 6.53	6.36
		1100	2273.66	107.27		
quercetin-3-*O*-glucoside	424.42	42.5	437.36	93.67	90.50 ± 3.94	4.35
		425	741.80	87.33		
quercetin-3-*O*-rutinoside	521.83	55	576.37	99.92	98.12 ± 5.27	5.37
		550	1032.49	96.33		
kaempferol-3-*O*-glucoside	177.95	17.5	177.82	90.98	90.37 ± 6.24	6.90
		175	316.81	89.76		

[a]Amount of each phenolic acid/flavonoid compound present originally in 0.5 g of rabbiteye blueberry leaf powder.
[b]Amount of each phenolic acid/flavonoid standard spiked into 0.5 g of rabbiteye blueberry leaf powder.
[c]Amount of each phenolic acid/flavonoid compound extracted and analyzed by UPLC-MS/MS from 0.5 g of rabbiteye blueberry leaf powder containing original content plus spiked standard.
[d]Recovery (%) = (amount of standard found - original amount in sample) / amount of standard spiked × 100.
[e]Relative standard deviation (%) = (standard deviation/mean) × 100.
